# Nutrigram^®^ as a novel BIA-derived parameter for assessing nutritional status in a cohort of post-stroke patients undergoing rehabilitation treatment

**DOI:** 10.3389/fnut.2026.1864371

**Published:** 2026-07-15

**Authors:** Chiara Bertoncini, Alessandro Guerrini, Carola Cocco, Mariacristina Siotto, Valeria Cipollini, Laura Cortellini, Arianna Pavan, Stefania Lattanzi, Dionysia Papadopoulou, Sabina Insalaco, Elisabetta Ruco, Erika Antonacci, Irene Giovanna Aprile

**Affiliations:** IRCCS Fondazione Don Carlo Gnocchi, Florence, Italy

**Keywords:** bioelectrical impedance analysis (BIA), malnutrition, Nutrigram^®^, nutritional screening, post-stroke rehabilitation

## Abstract

**Introduction:**

Early nutritional assessment is essential during post-stroke rehabilitation, which may require hospitalization lasting several months. Nutrigram^®^, a novel BIA-derived index, provides a rapid, objective, and non-invasive measure of nutritional status, but its role in post-stroke populations remains unexplored. This study aimed to evaluate the association between Nutrigram^®^, other established nutritional risk assessment tools, and malnutrition in subacute post-stroke patients.

**Methods:**

A total of 87 subacute post-stroke patients were evaluated at admission (T0). Demographic, clinical, and nutritional data were collected, and malnutrition was diagnosed using the Global Leadership Initiative on Malnutrition (GLIM) criteria. In the first GLIM step, nutritional risk screening was performed using both the Mini Nutritional Assessment Short Form (MNA-SF^®^) and the Geriatric Nutritional Risk Index (GNRI). Body composition was assessed by bioelectrical impedance analysis (BIA), which allowed for the recording of the Nutrigram^®^.

**Results:**

Nutrigram^®^ was significantly associated with both the MNA-SF^®^ and GNRI scores. Patients diagnosed as malnourished showed significantly lower Nutrigram^®^ values compared with non-malnourished patients, both after MNA-SF^®^ screening (582.7 ± 156.7 vs. 780.5 ± 208.9, *p* < 0.001) and GNRI screening (566.9 ± 162.3 vs. 749.0 ± 207.5, *p* < 0.001). Finally, Nutrigram^®^ was independently associated with malnutrition diagnosis, with higher values indicating lower odds of malnutrition.

**Conclusion:**

Nutrigram® appears to be a promising objective index of nutritional status in post-stroke patients, showing consistent associations with established screening tools and malnutrition diagnosis.

## 1 Introduction

Nutritional status assessment is an important aspect of healthcare in post-stroke survivors and is a systematic evaluation aimed at identifying malnutrition and the risk of malnutrition (1). Among post-stroke patients, malnutrition is a frequent and clinically relevant complication with a multifactorial etiology (2, 3). In fact, various post-stroke impairments—such as dysphagia, limited upper limb movements, visuospatial deficits, increased catabolic processes, and gastrointestinal dysfunction—can compromise adequate dietary intake and nutrient absorption, leading to malnutrition (1, 4–7). Early identification of malnutrition, along with timely implementation of appropriate interventions for at-risk patients, is essential to support recovery and enhance rehabilitation outcomes (8). For this purpose, the Global Leadership Initiative on Malnutrition (GLIM) framework provides a standardized, two-step diagnostic process designed to establish consensual criteria for malnutrition diagnosis (9). The first step consists of initial screening using validated tools to identify individuals at risk for malnutrition, while the second step involves confirmation of the diagnosis through the assessment of specific criteria (9).

In this context, the type of nutritional screening tool significantly influences the estimated prevalence of malnutrition in stroke patients, with reported rates varying depending on the instrument used. To date, no single tool has been definitively established as the most appropriate for detecting patients at risk of malnutrition following a stroke. However, as stated in a recent literature review, several nutritional screening tools are currently used among post-stroke survivors (8). Among these, the Mini Nutritional Assessment Short Form (MNA-SF®) and the Geriatric Nutritional Risk Index (GNRI) are the most widely used tools in rehabilitation settings (8).

Decreased MNA-SF® scores and GNRI values have been associated with unfavorable biochemical and hematological profiles, including lower albumin, hemoglobin, hematocrit, magnesium, calcium, and leptin levels, as well as increased homocysteine, C-reactive protein, resistin, and oxidative stress-related biomarkers in hospitalized older patients (10, 11). In addition, lower MNA-SF® scores and reduced GNRI values have been associated with poorer functional outcomes, including the Functional Independence Measure (FIM) (12), reduced independence in activities of daily living (ADL) (13), and a longer hospital stay in stroke survivors (14).

After the screening step, diagnostic confirmation requires the simultaneous fulfillment of at least one phenotypic criterion and one etiologic criterion (9). Weight loss, reduced BMI, and reduced muscle mass were categorized as phenotypic criteria, while reduced food intake/assimilation and disease burden/inflammation were categorized as etiologic criteria (9). Previous studies have demonstrated that malnourished post-stroke patients, according to GLIM, had lower functional recovery, as reflected by the FIM-motor score (15), and gain in the Modified Barthel Index, as well as lower food intake during the rehabilitation period (16).

In Italy, post-stroke rehabilitation may last several months and often involves prolonged hospitalization. In this context, early and accurate nutritional assessment, combined with targeted interventions, may significantly influence recovery. Therefore, rapid, objective, non-invasive, and easily interpretable methods are essential to identify patients at risk at admission to rehabilitation settings.

In this regard, the novel Nutrigram® parameter emerges as a potentially useful tool for nutritional assessment, as it fulfills all of these requirements. First, Nutrigram® is derived from bioelectrical impedance analysis (BIA), a widely used, safe, inexpensive, and non-invasive method for assessing body composition in post-stroke patients (17). Second, it provides an objective and comprehensive numerical index of nutritional status that is easy to interpret, with lower values indicating a higher risk of nutritional impairment, thus potentially facilitating its use in clinical settings (18). Finally, it can be evaluated in a few minutes, thereby enabling timely patient management and supporting the implementation of personalized interventions tailored to each patient’s specific nutritional requirements.

Specifically, Nutrigram® reflects changes in body cell mass, a proxy for the metabolically active cellular component of the body, which is directly involved in oxygen consumption and closely linked to nutritional status (18, 19). Previous studies in oncological populations demonstrated that lower Nutrigram® values were associated with underweight status, significant unintentional weight loss, and worsening functional status (18), as well as increased mortality rates and decreased tolerance to anticancer drugs (20).

Despite the high potential of Nutrigram® for assessing nutritional status, clinical evidence supporting its use in the post-stroke population is lacking.

To address this gap, we aim to investigate the association between Nutrigram® and established nutritional risk assessment tools (MNA-SF® and GNRI) in subacute post-stroke patients at admission to a rehabilitation center, and to evaluate their relationship with malnutrition as defined by the GLIM criteria. This analysis may provide preliminary evidence regarding the potential role of Nutrigram® in the nutritional assessment of post-stroke patients.

This study represents a secondary analysis of data from a clinical protocol originally designed to evaluate the potential correlation between nutritional status indices and rehabilitation outcomes in a cohort of post-stroke patients undergoing rehabilitation.

## 2 Materials and methods

### 2.1 Study design and participants

The study protocol, registered on ClinicalTrials.gov (brief title: NUTRISTROKE; identifier number: NCT04923165), is an observational multicenter longitudinal study designed to analyze the nutritional status of post-stroke patients admitted to two rehabilitation centers of Fondazione Don Carlo Gnocchi (Italy). Herein, we studied patients enrolled at the “*S. Maria* della Provvidenza” center located in Rome (RM, Italy) from September 2020 to April 2023. Patients were enrolled based on the following eligibility criteria: (i) first hemorrhagic or ischemic stroke documented by computed tomography (CT) or magnetic resonance imaging (MRI), (ii) age between 18 and 85 years, (iii) within 6 months of stroke onset, and (iv) sufficient cognitive and language abilities to provide informed consent and comprehend the assessment scales.

The exclusion criteria were the following: (i) a previous stroke, (ii) behavioral and cognitive disorders and/or reduced compliance interfering with active rehabilitation treatment or with the ability to understand and provide informed consent, and (iii) the presence of pacemakers (which may interfere with bioimpedance measures).

The study was conducted in accordance with the Declaration of Helsinki and approved by the Ethics Committee of Fondazione Don Carlo Gnocchi, Milan, on 12 February 2020, with a non-substantial amendment on 14 October 2020 (Prot.no.22/2020/CE_FdG/FC/SA_14/10/20). All patients provided written informed consent.

### 2.2 Rehabilitation treatment

Patients followed a 6-week rehabilitation program that included traditional physical therapies for 45 min per day, 6 days a week. Rehabilitation treatment included passive, active-assisted, and active mobilizations; exercises for restoring muscle strength; stretching; sensorimotor stimulation; postural transitions and transfers; proprioceptive exercises; motor coordination and balance training; walking, sitting, and standing training; and activities of daily living (e.g., transfers, dressing, and brushing/combing hair, depending on the subject’s ability) training.

Additionally, each patient received daily upper-limb robotic therapy, enriched with cognitive tasks, five times per week for 45 min per session, as detailed in previous studies (21, 22).

### 2.3 Clinical and nutritional assessment

Demographic, anamnestic, clinical, and anthropometric data were recorded at admission (T0). The comorbidities and disease severity were described according to the Cumulative Illness Rating Scale (CIRS) (23). The presence of dysphagia was recorded at T0 based on clinical evaluation.

Anthropometric measurements were assessed as follows: height was recorded for each patient who could stand, reporting data in meters (m). The height of patients who were unable to stand was measured with knee height, with the Chumlea equation (24). Body weight was reported in kilograms (kg), accurate to the nearest 0.1 kg. Patients able to stand were weighed on a calibrated scale (Seca 750, Seca, Hamburg, Germany); patients unable to stand were weighed on a chair weighing scale (Wunder DE5, Wunder Sa.Bi. srl; Milan, Italy). The BMI (kg/m^2^) was then calculated. Serum albumin concentration was measured, as detailed in a previous study by Cocco et al. (25).

#### 2.3.1 Assessment of the risk of malnutrition and diagnosis of malnutrition according to GLIM criteria

For the assessment of the risk of malnutrition, as well as the diagnosis of malnutrition, the latest GLIM criteria were applied (9). The risk of malnutrition was evaluated using two screening tools as the first step in the GLIM framework: the MNA-SF® and GNRI. The MNA-SF® is a quick and easy-to-use questionnaire, validated as a stand-alone screening tool, which consists of both objective components (e.g., body mass index and weight loss) and subjective elements based on patient-reported information (e.g., dietary intake, appetite, and perceived health status) (26). The GNRI is a biological index of nutritional risk status derived from serum albumin levels and ideal weight, and was calculated as follows: 14.89 × albumin (g/dL) + 41.7 × (weight/ideal body weight) (27). The cutoffs for malnutrition risk were defined as follows: MNA-SF® score <12 (28) and GNRI values < 98 (27). The first diagnostic procedure included patients at risk of malnutrition, as identified with the MNA-SF®, whereas the second included patients identified with the GNRI.

The next step in malnutrition diagnosis consists of identifying the presence of at least one phenotypic and one etiologic condition.

The phenotypic criteria adopted were as follows: (i) weight loss >5% within the past 6 months or >10% beyond 6 months; (ii) a low BMI, <20 if <70 years of age or <22 if >70 years of age; and (iii) reduced muscle mass assessed by calculating the Appendicular Skeletal Muscle Mass (ASMM) divided by the patient’s height squared (ASMM/h^2^; kg/m^2^), using a specific population- and BIA-validated predictive equation (29). The thresholds for reduced muscle mass were as follows: <7 kg/m^2^ for men and <5.5 kg/m^2^ for women. The etiologic criteria considered for our patients included the presence of inflammation due to acute disease, injury, or chronic disease. Since stroke is included among the conditions characterized by inflammation in the GLIM framework (30), the etiologic criterion was considered fulfilled in the entire cohort.

#### 2.3.2 Bioelectrical impedance analysis and Nutrigram®

Body composition was evaluated at admission to the rehabilitation center using a single-frequency bioelectrical impedance analysis device (BIA 101 Anniversary Sport Edition, Akern, Firenze, Italy), which applies an alternating sinusoidal electric current of 800 μA at a frequency of 50 kHz. The raw BIA parameters, resistance (*R_z_*) and reactance (*X_c_*), were subsequently obtained: *R_z_* reflects the opposition to the flow of an alternating electrical current, while *X_c_* is determined by the capacitance of the cell membranes (31).

All patients were examined in a supine position, with their limbs equally spaced. Because fluid shifts occur during the transition from standing to recumbency and directly affect *R_z_* and *Z* values, patients remained in this position for 5 min before measurements were taken. Before measurement, the skin at the contact points was cleaned to prevent alterations. Patients with fever or hypothermia were not analyzed because body temperature may alter bioelectric resistance. Patients’ hydration status was clinically assessed before the BIA evaluation, and patients presenting with local edema or severe dehydration were excluded. Additionally, patients with pacemakers were excluded. Operators were instructed to follow standardized data acquisition procedures before starting measurements to minimize estimation errors.

BIA was conducted using four certified electrodes (BIACTRODES, Akern, Florence, Italy), which were placed on the foot at the metatarsal site and on the hand at the metacarpal site of the hemisoma without hemiparesis, with a distance of 5 cm between adjacent electrodes.

Body composition parameters were estimated using predictive equations and regression analyses incorporating several variables, such as *R_z_*, *X_c_*, sex, age, stature, and weight. The BIA-derived parameters assessed in this study were the Appendicular Skeletal Muscle Mass Index (ASMMI) and Nutrigram®. Nutrigram® is a numerical index of nutritional status (18), calculated using the Bodygram Dashboard software (Version 3.4.14; Akern, Florence, Italy).

### 2.4 Statistical analysis

The sample size was established *a priori* for the primary outcome of the NUTRISTROKE protocol (NCT04923165). Specifically, G*Power software (version 3.1.9.7) was used, assuming an expected correlation of *r* = 0.3 between baseline nutritional indicators and functional recovery outcomes, a two-tailed *α* = 0.05, and a statistical power (1–*β*) of 0.80; the required sample size was estimated to be 85 participants. The results presented herein were derived from a secondary analysis of these data.

Demographic and clinical characteristics of the patients were presented as mean ± standard deviation for continuous variables and as counts and percentages for categorical variables. The normality of the data distribution was assessed using the Shapiro–Wilk test. Homogeneity of variances was verified with Levene’s test.

At the time of admission, demographic and clinical features along with nutritional status parameters of the sample were compared between women and men using the chi-squared test for categorical variables and the Mann–Whitney *U* test for non-parametric continuous variables.

Multiple linear regression analyses were then conducted to evaluate the association between risk of malnutrition, assessed by the MNA-SF® and GNRI, and Nutrigram® values (Model 1, unadjusted). To account for potential confounding factors that could influence these associations, adjusted models were subsequently performed (Model 2, adjusted). Covariates were selected based on clinical relevance and included age, sex, stroke type, presence of dysphagia, time from stroke onset, CIRS comorbidity index, and CIRS severity index. Preliminary bivariate analyses were conducted to explore the relationship between these variables and the GNRI and MNA-SF® scores. Spearman’s rank correlation coefficient or the Mann–Whitney U test was applied as appropriate. Variables showing a significant association in the bivariate analyses were then included, together with Nutrigram®, into the adjusted multiple linear regression models, with GNRI and MNA-SF® as dependent variables.

To investigate the potential association between Nutrigram® and malnutrition, as defined by the GLIM criteria (malnourished vs. non-malnourished), we first examined the relationship between the previously identified potential confounders and malnutrition status using bivariate analyses, applying Pearson’s chi-squared test or the Mann–Whitney *U* test, as appropriate.

Variables that showed significant differences between malnourished and non-malnourished patients in the bivariate analyses were subsequently included, together with Nutrigram®, in a multivariable logistic regression model, with malnutrition status as the dependent variable.

To enhance the clinical interpretability of the results, Nutrigram® values were rescaled by dividing the original values by 100 before linear and logistic regression analyses. Consequently, regression coefficients and odds ratios are expressed per 100 mg/24 h/m increase in Nutrigram®. Additionally, to assess the discriminative ability of Nutrigram® for identifying GLIM-defined malnutrition, receiver operating characteristic (ROC) curve analyses were performed, and the area under the ROC curve (AUC) and its 95% confidence interval were estimated.

For all statistical analyses, a *p*-value < 0.05 was considered statistically significant. Statistical analysis was performed using IBM SPSS Statistics (Version 28.0, Armonk, NY: IBM Corp.) and GraphPad Prism (version 11.0.1(90); GraphPad Software, San Diego, CA, USA).

## 3 Results

### 3.1 Participants’ clinical and nutritional characteristics at baseline

The complete baseline characteristics of the whole group and those stratified by sex have been published previously (25). In brief, we enrolled 91 patients; 4 patients were excluded due to adverse clinical conditions, and a final sample of 87 subacute post-stroke patients was analyzed.

Table 1 reports the baseline characteristics of the study population, including relevant data from the nutritional assessment. As expected, anthropometric measurements and body composition parameters differed significantly between women and men. No significant differences were found in serum albumin levels, MNA-SF® scores, and GNRI values between women and men.

**Table 1:** Baseline (T0) characteristics and nutritional status assessment were reported for the whole group (*n* = 87) and stratified by sex (female, *n* = 42; male, *n* = 45).

Baseline characteristics	Whole group (*n* = 87)	Women (*n* = 42)	Men (*n* = 45)	*p*-value
Age (years)	69 ± 12	71 ± 9	66 ± 13	0.110
Stroke type (ischemic)	66 (76%)	33 (79%)	33 (73%)	0.568
Days from stroke onset to enrollment	105 ± 52	114 ± 57	96 ± 47	0.136
Dysphagia	29 (33%)	16 (38%)	16 (30%)	0.363
Cumulative illness rating scale (CIRS)				
CIRS severity	2.3 ± 0.4	2.3 ± 0.3	2.3 ± 0.4	0.309
CIRS comorbidity	5.7 ± 1.7	5.7 ± 1.7	5.6 ± 1.8	0.570

Nutritional status assessment	Whole group (*n* = 87)	Women (*n* = 42)	Men (*n* = 45)	*p*-value
Anthropometric measurements				
Weight (kg)	69.6 ± 17.3	62.7 ± 13.9	75.9 ± 17.9	<0.001***
Height (m)	1.66 ± 0.11	1.61 ± 0.07	1.72 ± 0.11	<0.001***
BMI (kg/m^2^)	24.9 ± 4.9	24.3 ± 5.4	25.5 ± 4.4	0.058
Biochemical markers				
Serum albumin (g/dL)	3.8 ± 0.5	3.7 ± 0.5	3.9 ± 0.5	0.351
Malnutrition screening tools				
Mini nutritional assessment short-form (MNA-SF ®)	8 ± 2	7 ± 2	8 ± 2	0.592
Geriatric nutritional risk index (GNRI)	105 ± 13	104 ± 14	107 ± 11	0.292
Malnutrition assessment (GLIM)				
Screening – MNA-SF® (<12)	83 (95%)	40 (95%)	43 (96%)	0.944
Screening – GNRI (<98)	31 (36%)	18 (43%)	13 (29%)	0.174
GLIM diagnosis (after MNA-SF® screening)	37 (43%)	22 (52%)	15 (33%)	0.073
GLIM diagnosis (after GNRI screening)	26 (30%)	16 (38%)	10 (22%)	0.106
Bioelectrical impedance analysis (BIA)				
Nutrigram® [mg/24 h/m]	666.8 ± 225.3	563.5 ± 189.1	778.1 ± 209	<0.001***
Appendicular skeletal muscle mass (ASMM)	19.9 ± 6.9	15.8 ± 3.5	23.8 ± 7.1	<0.001***

Data are reported as mean ± standard deviation or number and percentage (%). *p*-values refer to the Mann–Whitney *U* test or the chi-squared test, as appropriate.

*p-value < 0.05, **p-value < 0.01, and ***p-value < 0.001.

**Figure 1:**
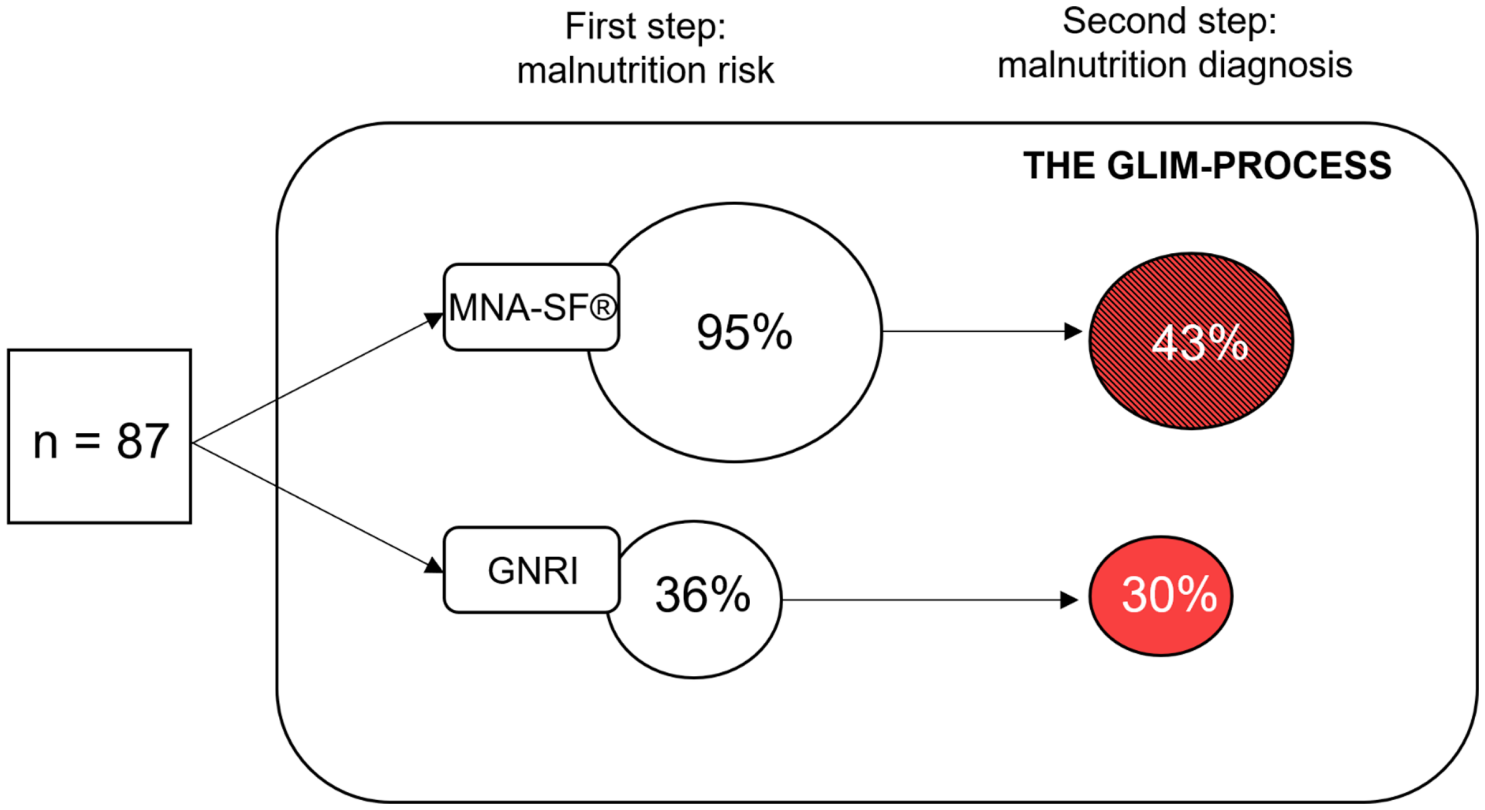
Results of the GLIM process shows the percentage of patients enrolled who were diagnosed with malnutrition when the Mini Nutritional Assessment Short-form (MNA-SF®) or the Geriatric Nutritional Risk Index (GNRI) was used as a screening tool.

We found differences in the prevalence of malnutrition among patients depending on which screening tool was used during the first step of the GLIM process. MNA-SF® identified a greater number of patients at risk of malnutrition than GNRI. Specifically, 83 post-stroke patients (95%, female patients *n* = 40) were classified as at risk of malnutrition according to MNA-SF®, whereas only 31 (36%, female patients *n* = 18) were identified as at risk using the GNRI cutoff (Figure 1).

When applying the GLIM criteria following MNA-SF® screening, malnutrition was confirmed in 37 post-stroke patients (43%, female patients, *n* = 22). In contrast, among the 31 post-stroke participants detected as at risk by GNRI, 26 were confirmed as malnourished (30%, female patients *n* = 16).

No significant differences in the prevalence of malnutrition diagnosis were observed between sexes using either screening approach.

### 3.2 Association between Nutrigram® and the risk of malnutrition

The associations between Nutrigram® and the MNA-SF® and GNRI scores, both unadjusted and adjusted for potential confounders, are presented in Tables 2, 3, respectively.

**Table 2:** Linear regression results for MNA-SF^®^.

MNA-SF^®^	B	95% CI		SE B	*p*-value	R2	ΔR2
		LL	UL				
Model 1 (unadjusted)						0.28	0.08
Nutrigram®	0.316	0.076	0.556	0.121	0.011		
Model 2 (adjusted)						0.34	0.12
Nutrigram®	0.264	0.021	0.507	0.122	0.034		
Presence of dysphagia	−1.001	−2.07	0.064	0.535	0.065		

B: unstandardized regression coefficient; CI: confidence interval; LL: lower limit; UL: upper limit; SE B: standard error of the coefficient; R2: coefficient of determination; ΔR2: adjusted R2. Nutrigram® was scaled by dividing by 100; unstandardized regression coefficients (B) are reported per 100 mg/24 h/m increase in Nutrigram®.

**Table 3:** Linear regression results for GNRI.

GNRI	B	95% CI		SE B	*p*-value	R2	ΔR2
		LL	UL				
Model 1 (unadjusted)						0.59	0.35
Nutrigram®	3.490	2.413	4.568	0.542	<0.001		
Model 2 (adjusted)						0.61	0.37
Nutrigram®	3.082	1.926	4.238	0.581	<0.001		
Presence of dysphagia	−2.77	−7.657	2.104	2.451	0.261		
Age	−0.143	−0.365	0.078	0.111	0.203		

B: unstandardized regression coefficient; CI: confidence interval; LL: lower limit; UL: upper limit; SE B: standard error of the coefficient; R2: coefficient of determination; ΔR2: adjusted R2. Nutrigram® was scaled by dividing by 100; unstandardized regression coefficients (B) are reported per 100 mg/24 h/m increase in Nutrigram®.

In the adjusted model, bivariate analyses showed no significant associations between sex, stroke type, time from stroke onset, CIRS comorbidity and severity, and either MNA-SF® or GNRI scores (Supplementary Table S1).

GNRI values were significantly lower in patients with dysphagia compared with those without dysphagia (100 ± 15 vs. 107 ± 11, *p* = 0.012). GNRI was also inversely correlated with age (Spearman’s rho = −0.340, *p* = 0.001). MNA-SF® scores were lower in patients with dysphagia than in those without; however, this difference did not reach statistical significance (7 ± 3 vs. 8 ± 2, *p* = 0.066). Based on these findings, dysphagia was included as a covariate in the adjusted model, whereas age was included only in the analysis examining the association with GNRI.

Nutrigram® was significantly associated with MNA-SF® in both unadjusted and adjusted models. A similar association was observed between Nutrigram® and GNRI.

### 3.3 Association between Nutrigram® and malnutrition diagnosis according to GLIM

**Figure 2:**
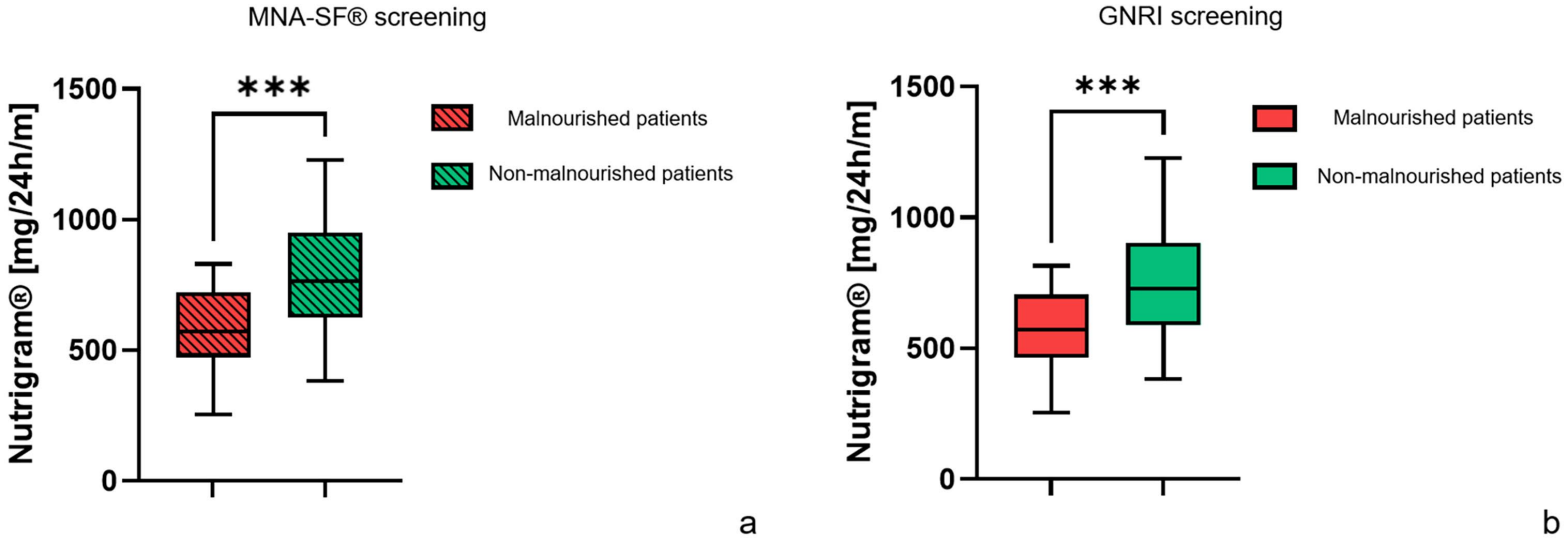
Box plots illustrate intergroup comparisons of Nutrigram® values (mg/24 h/mL) between malnourished and non-malnourished patients. **(a)** Comparison based on GLIM-defined malnutrition following MNA-SF® screening. **(b)** Comparison based on GLIM-defined malnutrition following GNRI screening. Median values, upper and lower quartiles, and minimum–maximum values are displayed for each group. ***Refer to the statistically significant differences (*p* < 0.001), according to the Mann–Whitney *U* test.

We observed significant differences in Nutrigram® values between patients with and without malnutrition, regardless of the screening tool used (Figure 1). In particular, patients diagnosed as malnourished following MNA-SF® screening exhibited significantly lower Nutrigram® values than non-malnourished patients (582.7 ± 156.7 vs. 780.5 ± 208.9, *p* < 0.001). Similar findings were observed when GNRI was used as the screening tool, with confirmed malnourished patients showing lower Nutrigram® values than non-malnourished patients (566.9 ± 162.3 vs. 749.0 ± 207.5, *p* < 0.001) (Figure 2).

Differences between malnourished post-stroke patients and non-malnourished ones were also observed regarding age and the prevalence of dysphagia, regardless of the screening approach applied (Supplementary Table S2). In contrast, no significant differences were observed for sex, stroke type, time since stroke onset, and CIRS comorbidity and severity between malnourished and non-malnourished groups (Supplementary Table S2). Consequently, age and dysphagia were included as covariates in the multivariable regression analyses. These models confirmed that Nutrigram® was independently associated with malnutrition diagnosis (Tables 4, 5), with higher Nutrigram® values associated with lower odds of malnutrition. ROC curve analyses further supported these findings, showing good discriminative ability of Nutrigram® in both subgroups: AUC = 0.762 (95% CI: 0.660–0.865, *p* < 0.001) for identifying GLIM-defined malnutrition among patients screened with the MNA-SF®, and AUC = 0.741 (95% CI: 0.623–0.852, *p* < 0.001) among those screened with the GNRI (Supplementary Figures S1, S2).

**Table 4:** Multiple logistic regression analysis for the diagnosis of malnutrition (after MNA-SF^®^ screening).

Malnutrition (GLIM criteria)	B	SE	*p*-value	*Z*	OR	95% CI (OR)	
						LL	UL
Nutrigram®	−0.527	0.161	**0.001**	−3.279	0.590	0.431	0.809
Presence of dysphagia	0.582	0.545	0.286	1.067	1.789	0.614	5.212
Age	0.011	0.026	0.668	0.428	1.011	0.960	1.065

B: unstandardized regression coefficient; SE, standard error of the coefficient; OR, odds ratio; 95% CI (OR), 95% confidence interval (odds ratio scale). Nutrigram® was scaled by dividing 100; ORs are reported per 100 mg/24 h/m increase in Nutrigram®. *p-value < 0.05, **p-value < 0.01, and ***p-value < 0.001.

**Table 5:** Multiple logistic regression analysis for the diagnosis of malnutrition (after GNRI screening).

Malnutrition (GLIM criteria)	B	SE	*p*-value	*Z*	OR	95% CI (OR)	
						LL	UL
Nutrigram®	−0.435	0.167	**0.009**	−2.600	0.647	0.466	0.898
Presence of dysphagia	0.994	0.562	0.077	1.769	2.702	0.898	8.129
Age	0.033	0.030	0.283	1.074	1.033	0.973	1.097

B: unstandardized regression coefficient; SE, standard error of the coefficient; OR, odds ratio; 95% CI (OR), 95% confidence interval (odds ratio scale). Nutrigram® was scaled by dividing by 100; ORs are reported per 100 mg/24 h/m increase in Nutrigram®. *p-value < 0.05, **p-value < 0.01, and ***p-value < 0.001.

## 4 Discussion

To the best of our knowledge, this is the first study to investigate the potential usefulness of Nutrigram®, a novel BIA-derived nutritional parameter, for assessing nutritional status in a cohort of post-stroke patients admitted to a rehabilitation center. Nutrigram® values were independently associated with both the MNA-SF® and GNRI scores during admission. In addition, patients diagnosed with malnutrition according to GLIM criteria exhibited significantly lower Nutrigram® values than non-malnourished individuals, regardless of the screening tool used. Finally, higher Nutrigram® values were independently associated with lower odds of malnutrition.

In this study, the nutritional status of subacute post-stroke patients admitted to our rehabilitation center was evaluated by assessing both the risk of malnutrition and the diagnosis of malnutrition according to the latest GLIM criteria, using a two-step approach (9). In the first step, both MNA-SF® and GNRI were used for malnutrition risk screening. These two tools were selected for several reasons. Recent evidence has identified the MNA-SF® and the GNRI among the most reliable screening tools in post-stroke survivors (8), as they have also demonstrated prognostic value for cognitive impairment (12, 32, 33), independence in ADL (13), poor functional outcomes (12, 34), mortality (35), and infectious diseases (14). At the same time, previous reviews have concluded that a single method is insufficient for accurately determining nutritional status (36, 37), and the literature has not reached consensus on a single optimal tool for identifying patients at risk of malnutrition following stroke (1). Given the above, the selection of both the MNA-SF® and the GNRI appears to be a justified and appropriate approach for screening malnutrition risk in this population.

However, methodological heterogeneity across nutritional screening and assessment tools can lead to substantial variability in the reported prevalence of malnutrition, even among similar patient populations (11, 38). In older inpatients, prevalence estimates have ranged from 30 to 90%, depending on the tool used to assess nutritional status (11).

In the present study, the use of the two screening tools influenced the final prevalence of confirmed malnutrition. Specifically, nearly all subjects were identified by MNA-SF®, whereas GNRI identified only approximately one-third of patients at risk. These discrepancies may be explained by differences in the methodological approaches of the screening tools. In particular, MNA-SF® includes items such as mobility, neuropsychological status, and acute disease, which may be more strongly influenced by stroke-related impairments rather than nutritional status itself (39). In contrast, the GNRI is derived from measured physiological parameters that are less influenced by neurological impairment, although serum albumin may still be affected by acute-phase responses (40).

Regardless of the screening tool or diagnostic framework used, the assessment of nutritional status remains crucial in post-stroke patients, as both malnutrition risk and established malnutrition have been consistently associated with poorer functional outcomes during rehabilitation (8, 16, 41). Moreover, given that the post-stroke hospitalization for rehabilitation may extend for months, the early identification of nutritional impairments at admission, as well as accurate monitoring of nutritional status during rehabilitation treatment, is crucial.

In this context, the use of rapid, objective, non-invasive, and easy-to-interpret nutritional tools is important. For these reasons, the new BIA-derived Nutrigram® appears to be a potentially useful nutritional tool, as it meets all of these requirements.

Furthermore, Nutrigram® may offer additional advantages over the previously mentioned tools for nutritional status assessment. Specifically, unlike the MNA-SF®, Nutrigram® is entirely objective and operator-independent, thereby minimizing the risk of subjectivity-related bias. In contrast to GNRI, Nutrigram® does not require biochemical analysis, thus avoiding the need for blood sampling. Additionally, because Nutrigram® provides a continuous numerical index rather than a categorical classification, it may offer supplementary information on longitudinal changes in nutritional status that the categorical structure of the GLIM criteria cannot capture. Despite its potential usefulness, evidence on the application of Nutrigram® in post-stroke populations is currently lacking. To date, its clinical utility has been mainly explored in the cancer population (18, 20).

In this study, we demonstrated that Nutrigram® was associated with both the MNA-SF® and GNRI scores. Its clinical relevance extended to the assessment of established malnutrition. Indeed, differences in Nutrigram® values were observed between patients with and without malnutrition, regardless of the screening tool used. Specifically, patients classified as malnourished according to GLIM showed lower Nutrigram® values compared with non-malnourished individuals. In multivariable analyses, higher Nutrigram® values remained independently associated with lower odds of GLIM-defined malnutrition. The discriminative ability of Nutrigram® was further supported by ROC curve analyses, which demonstrated good performance in both screening subgroups, suggesting that this parameter may provide clinically useful and complementary information for identifying patients with confirmed malnutrition in the post-stroke rehabilitation setting.

In our view, the findings derived from the Nutrigram® analysis are consistent with the clinical and nutritional characteristics typically observed in post-stroke patients. Specifically, Nutrigram® has been developed to reflect the metabolically active component of lean soft tissue through a model based on estimated 24-h creatinine production (18). In the presence of malnutrition, lean soft tissues, including skeletal muscle mass, are progressively mobilized to meet energy and metabolic demands, leading to their wasting over time (1, 42–46). This process is particularly relevant in post-stroke patients, who commonly experience a marked loss of muscle and lean mass (47). Consistent with this, in our previous study, we demonstrated that malnourished post-stroke patients exhibited lower levels of lean soft tissue than their well-nourished counterparts (46). This difference may explain the lower Nutrigram® values observed in malnourished post-stroke patients in the present study.

Considering the above, our findings suggest that Nutrigram® assessment at the beginning of rehabilitation treatment may contribute to a more comprehensive evaluation of nutritional status in post-stroke patients, thereby enabling prompt nutritional interventions that can mitigate functional decline (48), reduce complications (49), and shorten hospital stay (1).

This study has several limitations. First, patients were recruited from a single rehabilitation center, which limits the generalizability of the findings. However, a multicentre study (trial registered at ClinicalTrials.gov under the identifier NCT06547827) will allow us to analyze more precisely the association between malnutrituion risk and rehabilitation outcome.

Another limitation is that Nutrigram® is based on an equation developed by the manufacturer and is therefore specific to a particular bioelectrical impedance analysis device. As a result, its applicability is limited to the BIA device used in this study. A further limitation concerns potential methodological overlap between Nutrigram® and the GLIM diagnostic procedure, given their shared BIA-based framework. Future studies should consider using an independent method, such as DXA, to assess the low muscle mass criterion in GLIM, thereby allowing a more independent evaluation of the association with Nutrigram®. Further studies in larger and more heterogeneous cohorts are warranted to confirm these findings and to better define the clinical relevance of Nutrigram®, potentially enhancing its utility in routine post-stroke care.

## 5 Conclusion

Nutrigram® appears to be a promising, objective, rapid, and easy-to-interpret index of nutritional status in post-stroke patients, showing consistent associations with established screening tools and with the diagnosis of malnutrition.

## Data Availability

The data supporting the findings of this study are available from the corresponding author upon reasonable request.
