# Supplementary material for: Nutrigram® as a novel BIA-derived parameter for assessing nutritional status in a cohort of post-stroke patients undergoing rehabilitation treatment
Source: Front Nutr. 2026 Jul 15;13:1864371. doi: 10.3389/fnut.2026.1864371 (PMC13414266; doi:10.3389/fnut.2026.1864371)
Supplement: Supplementary file 1 [file Data_Sheet_1.PDF]

Supplementary Table 1. Bivariate analyses of the relationship between clinical and demographic variables and MNA-SF® and GNRI scores. Continuous variables were analysed using Spearman's rank correlation coefficient (ρ), while categorical variables were compared using the Mann–Whitney U test. Continuous data are expressed as mean ± standard deviation.

**Variables**	**MNA-SF®**	***p-Value***	**GNRI**	***p-Value***
Age (years)	ρ = − 0.019	0.858	ρ = − 0.340	0.001
Sex				
Women	8 ± 2	0.592	106 ± 11	0.292
Men	7 ± 2		103 ± 14	
Stroke Type				
Ischemic patients	7 ± 2	0.370	105 ± 13	0.740
Hemorrhagic patients	8 ± 2		104 ± 13	
Days from stroke onset to enrolment	ρ = 0.086	0.428	ρ = − 0.043	0.690
Presence of dysphagia				
Dysphagic patients	7 ± 2	0.066	100 ± 15	0.012
Non-dysphagic patients	8 ± 2		107 ± 11	
Cumulative Illness Rating Scale (CIRS)				
CIRS severity	ρ = − 0.027	0.803	ρ = 0.061	0.574
CIRS comorbidity	ρ = 0.046	0.671	ρ = 0.062	0.571

Supplementary Table 2. Bivariate analyses comparing clinical and demographic variables between malnourished and non-malnourished patients, as defined by GLIM criteria following MNA-SF® or GNRI screening. Continuous variables were compared using the Mann–Whitney U test, while categorical variables were analyzed using the chi-squared test. Data are expressed as mean ± standard deviation for continuous variables and as count and percentage for categorical variables.

**Variables**	**GLIM-defined malnutrition**					
	MNA-SF® screening			*GNRI-SF® screening*		
	Malnourished n = 37	*Non-malnourished* n = 50	*p-Value*	Malnourished n = 26	*Non-malnourished* n = 61	*p-Value*
Age (years)	72 ± 9	66 ± 13	0.039	73 ± 9	66 ± 12	0.015
Sex (Women)	22 (59%)	20 (40%)	0.073	16 (61%)	26 (43%)	0.106
Stroke Type (ischemic)	29 (78%)	37 (74%)	0.637	18 (69%)	*48 (79%)*	*0.345*
Days from stroke onset to enrolment	104 ± 46	97 ± 57	0.160	108 ± 48	96 ± 54	0.131
Presence of dysphagia	17 (46%)	12 (24%)	0.032	14 (53%)	15 (25%)	0.008
Cumulative Illness Rating Scale (CIRS)						
CIRS severity	2.3 ± 0.3	2.3 ± 0.4	0.394	2.3 ± 0.3	2.3 ± 0.4	0.852
CIRS comorbidity	5.7 ± 1.5	5.6 ± 1.9	0.517	5.7 ± 1.6	5.7 ± 1.8	0.970

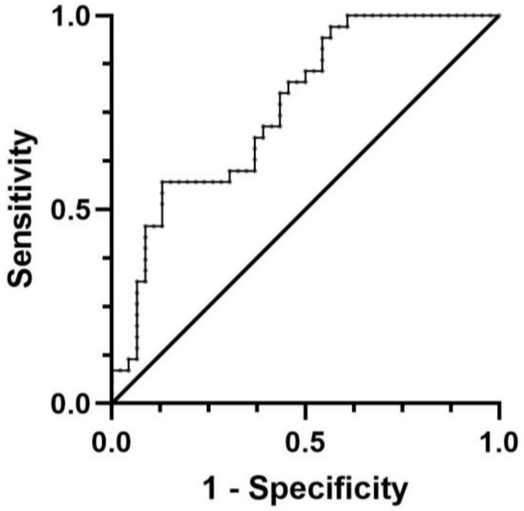

Supplementary figure 1. Receiver Operating Characteristic (ROC) curve of Nutrigram® for detecting GLIM-defined malnutrition following MNA-SF® screening in post-stroke patients. AUC 0.762, 95%CI 0.660-0.865, p<0.001.

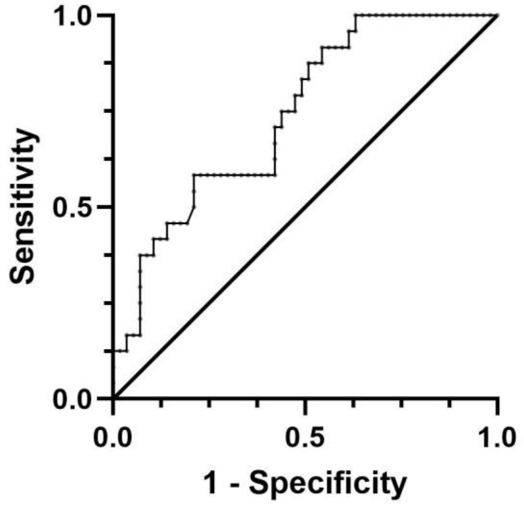

Supplementary figure 2. Receiver Operating Characteristic (ROC) curve of Nutrigram® detecting GLIM-defined malnutrition following GNRI screening in post-stroke patients. AUC 0.741, 95%CI 0.623-0.852, p<0.001.
